# Application of Bayesian networks to the prediction of the AMEn: a new methodology in broiler nutrition

**DOI:** 10.1093/tas/txaa215

**Published:** 2021-01-22

**Authors:** Tatiane C Alvarenga, Renato R Lima, Júlio S S Bueno Filho, Sérgio D Simão, Flávia C Q Mariano, Renata R Alvarenga, Paulo B Rodrigues

**Affiliations:** 1 Department of Statistics, Federal University of Lavras, Lavras, Minas Gerais, Brazil; 2 Department of Animal Science, Federal University of Lavras, Lavras, Minas Gerais, Brazil; 3 Department of Science and Technology, Federal University of São Paulo, São José dos Campos, São Paulo, Brazil

**Keywords:** graph models, max-min hill-climbing algorithm, metabolic energy, probability distributions

## Abstract

Designing balanced rations for broilers depends on precise knowledge of nitrogen-corrected apparent metabolizable energy (AMEn) and the chemical composition of the feedstuffs. The equations that include the measurements of the chemical composition of the feedstuff can be used in the prediction of AMEn. In the literature, there are studies that obtained prediction equations through multiple regression, meta-analysis, and neural networks. However, other statistical methodologies with promising potential can be used to obtain better predictions of energy values. The objective of the present study was to propose and evaluate the use of Bayesian networks (BN) to the prediction of the AMEn values of energy and protein feedstuffs of vegetable origin used in the formulation of broiler rations. In addition, verify that the predictions of energy values using this methodology are the most accurate and, consequently, are recommended to Animal Science professionals area for the preparation of balanced feeds. BN are models that consist of graphical and probabilistic representations of conditional and joint distributions of the random variables. BN uses machine learning algorithms, being a methodology of artificial intelligence. The bnlearn package in R software was used to predict AMEn from the following covariates: crude protein, crude fiber, ethereal extract, mineral matter, as well as food category, i.e., energy (corn, corn by-products, and others) or protein (soybean, soy by-products, and others) and the type of animal (chick or cockerel). The data come from 568 feeding experiments carried out in Brazil. Additional data from metabolic experiments were obtained from the Federal University of Lavras (UFLA) – Lavras, Minas Gerais, Brazil. The model with the highest accuracy (mean squared error = 66529.8 and multiple coefficients of determination = 0.87) was fitted with the max-min hill climbing algorithm (MMHC) using 80% and 20% of the data for training and test sets, respectively. The accuracy of the models was evaluated based on their values of mean squared error, mean absolute deviation, and mean absolute percentage error. The equations proposed by a new methodology in avian nutrition can be used by the broiler industry in the determination of rations.

## INTRODUCTION

Production of low-cost high protein chicken meat through intensively reared broiler chickens has high economic importance at national and international levels. The need to formulate diets that are increasingly adequate to the demands of broilers is necessary for the production system. The productive efficiency of birds is directly related to the adequate supply of dietary energy, which, in turn, depends on the nitrogen-corrected apparent metabolizable energy (AMEn) of the foods. However, one of the highest problems actually is the real knowledge of the energy composition of feedstuffs, which directly interferes with the energy levels of the rations and, consequently, on the nutrient balance of the same. Currently, several methods are available to assess the energy composition of feedstuffs and, often, discrepant results are observed.

The energy values feedstuffs can be obtained in biological tests, with the execution is time-consuming and of high cost, or by the composition tables of the feedstuffs (Albino, 1980). Another way of obtaining the values of AMEn is the prediction equations established according to the chemical composition of the feedstuffs, which is usually easy and quick to obtain (Rodrigues et al., 2001, 2002). Zhao et al. (2008) developed prediction equations using multiple regression to estimate the energy values using the chemical composition of the feedstuffs; however, their results have been inconsistent or applicable only to one feedstuff group (Alvarenga et al., 2011). Nascimento et al. (2009, 2011) and Mariano et al. (2012) used meta-analyses to better predict AMEn. Perai et al. (2010), Ahmadi et al. (2007, 2008), and Mariano et al. (2013) used neural networks (NN), and the latter used a larger number of foods and in vivo trials.

NN and Bayesian networks (BN) are suitable tools for prediction due to their superior ability to capture and express complex dependencies on covariates and response variables (Bishop, 2006; Gianola et al., 2011). BN has been used in medicine, genetics, robotics, economics, demography forensics, education, human behavior, industrial applications, species conservation, and mining (Pourret et al., 2008). Mariano et al. (2013) focused on predicting AMEn using a NN. Felipe et al. (2015) indicated the possibility of using BN in Animal Science; however, the previous use of BN for Animal Science papers is not restricted to breeding and genomic selection (Gianola et al., 2011; Morota et al., 2013). These approaches have not yet been applied to examine broiler nutrition.

To find more accurate results, BN are used to predict the AMEn according to the chemical composition of feedstuffs, BN are graphical models, which consist of the graphical representation (graph) and probabilistic (conditional and joint probability distributions) of the variables (Scutari and Denis, 2015; Koller and Friedman, 2009; Lauritzen and Spiegelhalter, 1988; Spirtes et al., 2000). In the applied areas, mainly Agriculture, there are still very few publications, however, Bayesian networks are an unprecedented line of research in poultry nutrition and that can be studied by researchers who are interested in predicting the values of metabolizable energy (Alvarenga et al., 2020).

Among the benefits of using BN are: 1) reducing the costs of in vivo trials to determine AMEn values, 2) Enhancing the accuracy of predictions of AMEn, 3) Reducing the variability in tabulated values for AMEn, 4) Expanding the use of Bayesian networks to areas where machine learning and related methods are starting to be employed, and 5) Capturing conditional dependency among random variables in, a broader sense than traditional methods can achieve. In this paper, the proposal using and evaluate BN, a new methodology in broiler nutrition, to obtain prediction equations for AMEn from a meta-analysis of energy and protein feedstuffs used for determining broiler rations.

## MATERIALS AND METHODS

### Data

To obtain the equations via BN, data from the meta-analysis were used, referring to the experiments conducted in Brazil in the period from 1967 to 2007, resulting in 568 experiments (Nascimento et al., 2009; Nascimento et al., 2011), among them which refer to the values of AMEn and chemical composition of energy (*n* = 370) and protein (*n* = 198) feedstuffs, of vegetable origin, commonly used in the formulation of broiler diets. The data used to validate the proposed equations were obtained by Alvarenga et al. (2011). These data come from two in vivo trials to determine the energy value of protein and energy feedstuffs, with growing chicks (traditional method of total excreta collection), respectively in February/March and July 2008. The trials were carried out in Lavras, state of Minas Gerais, Brazil (21° 14′ 45″S, 44° 59′ 59″W, 919 m a.s.l.) at the Federal University of Lavras (Alvarenga et al., 2011). For both data used to obtain and validate the equations via BN, the values of the response variable – AMEn, were estimated by the covariables; crude protein (CP), ether extract (EE), ash, crude fiber (CF), classification of the feedstuffs category (1 – energy concentrate, 2 – protein concentrate), specification of the ingredient in the category (1 – energy concentrate): (1 – corn, 2 – corn by-products, 3 – others), the ingredient specification in the category (2 – protein concentrate): (1 – soybean, 2 – soybean by-products, 3 – others) and the type of animal used in the bioassay (1 – chicks, 2 – cockerels).

### Prediction Models

The structure of a directed acyclic graph (DAG) that represents the BN, the nodes are connected, and all the arrows are directed without cycling (the arrow cannot return to the same node). The DAG is a directional, connected, and acyclic graph. We can observe that the neighbors of a node are the adjacent nodes, which are either parents or sons (Nagarajan et al., 2013).

Most algorithms used to find graph structure depend on topology because causal relations are associated with precedence for conditioning. Some of the algorithms use a Markov blanket to the target node. The nodes that separate the target node from the remaining structure are parent, child, and nodes that share a child with the target node. For prediction, only those variables would be relevant to modeling (Koski and Noble, 2009; Scutari and Denis, 2015).

A BN is a graphic representation of a joint probability distribution (or joint density, Margaritis, 2003). It can be described by the structure of a DAG. Factorization of the BN, as described by equation 1, is a chain of products of conditional probabilities, as one node, given its parents, is conditionally independent of its non-descendants (Pearl, 1988; Koski and Noble, 2009; Scutari and Denis, 2015). This is a convenient representation of the joint probability distribution, allowing for an inference on the desired research questions. The joint probability distribution is defined as:

$$\begin{array}{l} P\left(X_{1},X_{2},...X_{p}\right)=\prod_{i=1}^{n}P\;\left(X_{i}|\;Pa_{i}\right), \end{array}$$ (1)

where *p* is the number of variables, *i* is the counter of samples and *n* is the number of observations. For the case of discrete and continuous nodes in which Pa_*i*_ are the parents of *X*_*i*._

The variables used to learn the DAG were CP, EE, ash, CF, food category (1 – energy concentrate, with ingredients: 1.1 – corn, 1.2 – corn by-products, 1.3 – others, 2 – protein concentrate, with ingredients 2.1 – soybean meal, 2 – soybean by-products, 3 – others) and type of animal used in the bioassay with two levels: 1 – chicks, 2 – cocks).

The initial step for a BN is to have an algorithm to learn the basic graph structure (Scutari, 2010). The next step is to learn the implicit local distributions for this given structure (Scutari et al., 2014). Nagarajan et al. (2013) discussed three algorithms for learning network structure. The first, constraint-based algorithms, are based on conditional independence tests to infer the arrow direction between nodes. The second, score-based algorithms, select among all possible structures the BN with the highest quality, scored by probability-based measures such as Akaike information criterion (AIC) or Bayesian (Schwarz) information criterion (BIC). The third type, hybrid algorithms, combine ideas of both.

The bnlearn package (Scutari, 2010; R Core Team, 2020) for R implements the following constraint-based algorithms: Grow-Shrink (GS) incremental association Markov blanket (IAMB) fast incremental association (Fast-IAMB) interleaved incremental association (Inter-IAMB); max-min parents and children (MMPC); semi-interleaved Hiton-PC (SI-HITON-PC). Each can be used for conditional independence tests (Nagarajan et al., 2013).

The score-based algorithms also implemented are hill climbing (HC) (Margaritis, 2003) and Tabu search (TABU). The scoring function can be AIC, BIC, or others. Hybrid algorithms include max-min hill climbing (MMHC) (Tsamardinos et al., 2006) and general 2-phase restricted maximization (RSMAX2). MMHC uses constraint-based MMPC to search graph skeletons, estimating parent–child Markov coverage for each pair of variables in BN. To determine directionality, a score-based HC algorithm is used. A more general implementation of MMHC is performed by the RSMAX2 algorithm. It can use any combination of constraint-based and score-based algorithms (Scutari and Denis, 2015).

AMEn predictions were performed using a hybrid BN with continuous and discrete variables in the same fashion as a multiple linear regression model (Koski and Noble, 2009). To envision the process, consider a set *X* of random variables, partitioned into two subsets: *X*_*D*_ for discrete variables and *X*_*C*_ for continuous variables. The joint probability distribution for *P*(*X*) can be factorized as:

$$\begin{array}{ll} P(X)= & \;P(X_{D},X_{C})\\ & =\prod_{i\;\in \;D}P(X_{i}|Pa_{D})\prod_{i\;\in \;\;C}P(X_{j}|Pa_{D},Pa_{C}), \end{array}$$

in which *Pa*_*D*_ and *Pa*_*C*_ are joint probabilities for each of the subsets, respectively.

The term $\prod_{i\;\;\in \;\;C}P(X_{j}||Pa_{D},Pa_{C})$ brings both discrete and continuous parent variables that can be locally represented by linear regressions with parameters from discrete parents. This is equivalent to writing:

$$\begin{array}{ll} & (X_{j}|Pa_{D},Pa_{C})∼(N\mu_{j},\sigma^{2}\;\;{\;}_{X_{j}|Pa_{D}}),\;in\;which\;\\ & \mu_{j}=\beta_{0,X_{j}|Pa_{D}}+\beta_{i,X_{j}|Pa_{D}}X_{{}_{j}|Pa_{c}}. \end{array}$$

Thus, for the prediction of AMEn, $\mu_{j}$ refers to the intercept for each level of the discrete variable's combination (categories for food and animal types). $\beta_{0,X{\;}_{j}|Pa_{D}}$ and $\beta_{i,X{\;}_{j}|Pa_{D}}$ are the intercept and coefficients of the multivariate linear regression, respectively. $X_{i|Pa_{C}}$ represents the variables CP, ash, EE, and CF.

The original data were described by Mariano et al. (2013). For this study, the data were randomly partitioned into a training set (80% of the sample size) and a testing set (using the remaining data). The training set was used to search for a best-fitted DAG. Equations derived from the joint posterior were compared to a metabolic data assay from Alvarenga et al. (2011). The parameters used for the validation of the model were simple correlation coefficient (*r*), multiple coefficients of determination (*R*^2^), mean squared error (MSE), mean absolute deviation (MAD), mean absolute percentage error (MAPE), bias (bias) (Mariano et al., 2014) and prediction mean squared error (PMSE) (Felipe et al., 2015).

## RESULTS

**Figure 1.**
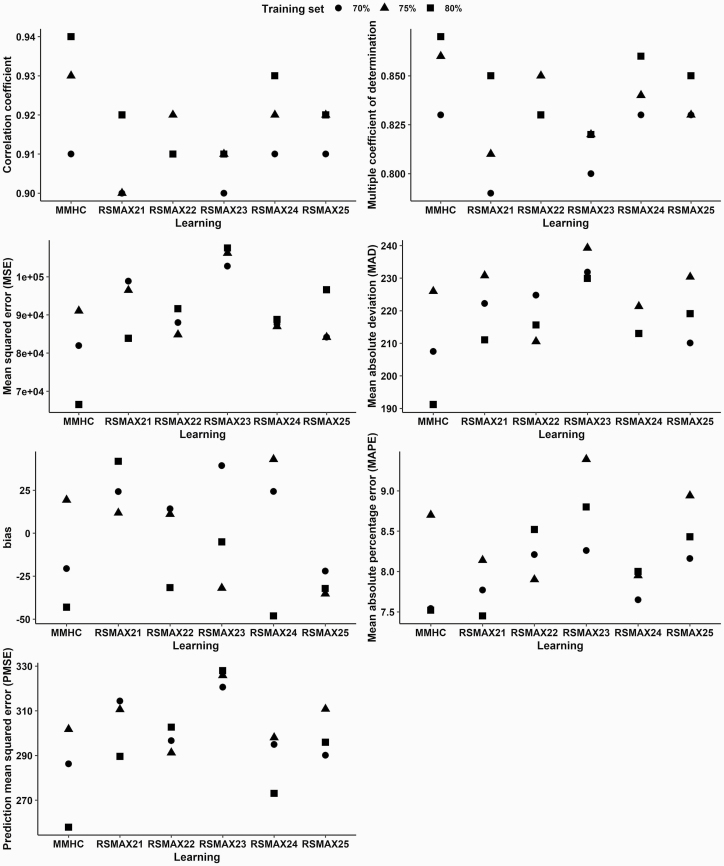
Evaluation of the performance and accuracy of the models for nitrogen-corrected apparent metabolizable energy (AMEn) estimated using Bayesian networks. *r*: correlation coefficient; *R*^2^: multiple coefficient of determination; MMHC: max-min hill climbing; RSMAX2: 2-phase restricted maximization; Learning respectively, constraint-based and score-based: 1 – semi-interleaved Hiton Parents and Children (SI.HITON.PC) and hill climbing (HC); 2 – interleaved incremental association (INTER.IAMB) and HC; 3 – fast incremental association (FAST.IAMB) and HC; 4 – incremental association Markov blanket (IAMB) and HC; 5 – Grow-Shrink (GS) and HC.

Different hybrid structures learning algorithms were evaluated, obtained from randomizations in the training data sets (80%, 75%, and 70%) and test (20%, 25%, and 30%). The best result obtained was through the MMHC learning algorithm (Figure 1) with the randomization of 80% of the learning data compared to the sets of 70% and 75%. The fit statistics were: *r* = 0.94, *R*^2^ = 0.87, MSE = 66529.8, MAD = 191.2, MAPE = 7.52, bias = −43.09 and PMSE = 257.93. The selected algorithm MMHC provided better statistics, except for RSMAX21 learning (MAPE = 7.45), with a difference of approximately 1%, and RSMAX24 (bias = −48.10), presenting a difference of approximately 10% (Figure 1).

**Figure 2.**
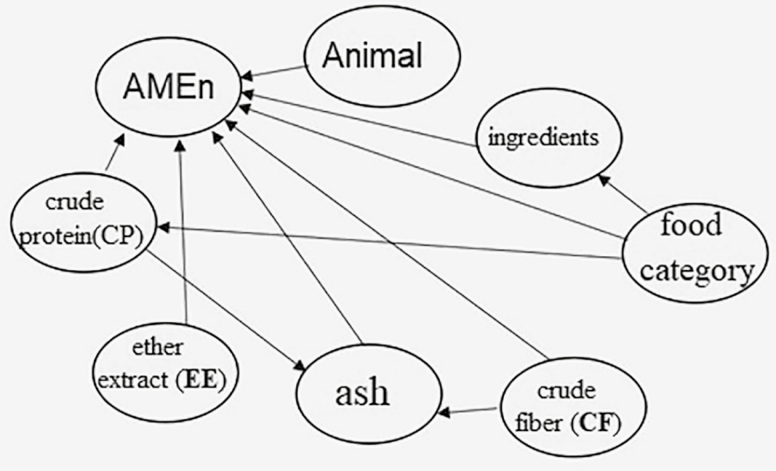
Directed acyclic graph (DAG) for study variables.

Table 1 summarizes the training (80% of the data) and testing sets. The DAG with the best-fitting yield by the MMHC learning algorithm is depicted in Figure 2, according to the result of the BN model presented in Figure 1. It has eight nodes and 11 arrows in a Markov blanket with seven nodes. The best learning algorithm was MMHC, i.e., using a constraint-based MMPC algorithm with conditional independence testing using mutual information. The scored-based method was hill climbing, using the BIC criterion. The number of tests used to learn the best DAG was 165.

**Table 1.** Summaries for variables in the training (80%) and testing (20%) sets. Original data from Nascimento et al. (2009, 2011)

	Statistics	AMEn (kcal/kg)	CP (%)	EE (%)	Ash (%)	CF (%)
Training set						
	Minimum	1,170	1.470	0.030	0.300	0.020
	Median	3,501	14.130	3.480	2.110	3.020
	Mean	3,176	23.360	4.872	3.560	4.928
	Maximum	4,386	71.440	26.210	12.610	26.500
Testing set						
	Minimum	1,148	1.700	0.030	0.560	0.320
	Median	3,275	15.270	3.150	3.010	3.985
	Mean	3,050	23.360	4.135	3.826	5.683
	Maximum	4,160	68.810	25.540	11.050	27.630

AMEn: nitrogen-corrected apparent metabolizable energy; CP: crude protein; EE: ether extract; CF: crude fiber.

The joint distribution represented in Figure 2 can be written as P(AMEn, CP, EE, ash, CF, Category, Ingredient, Animal) = P(EE) · P(CF) · P(Category) · P(Animal) · P(CP | Category) · P(Ingredient | Category) · P(ash | CP:CF) · P(AMEn | CP : EE : ash : CF : Category : Ingredient : Animal). This means that EE, CF, Category, and Animal are not dependent on the other variables; however, CP is dependent on Category, and ash is dependent by CP and CF. The response variable AMEn is conditionally dependent on all studied variables. Thus, there are 12 regression equations to AMEn, each coming from a different combination of levels for the discrete variables. Each separate prediction equation uses only levels of quantitative variables (CP, CF, ash, and EE). The proposed prediction equations and their coefficients are presented in Table 2.

**Table 2.** Prediction equations for nitrogen-corrected apparent metabolizable energy (AMEn) estimated using Bayesian networks

AMEn (kcal/kg)			Coefficients				
Category	Animals	Type food	Intercept	CP	EE	Ash	CF
Energy	Chicks	Corn	3658.16	−2.41	−11.25	+83.41	+ 16.76
		Corn by-products	4209.57	−34.56	+32.84	−25.15	−142.57
		Other Corn products	4335.88	−50.91	+35.40	−67.35	−87.06
Protein	Chicks	Soybean	3684.83	−19.84	−71.15	+18.14	−8.93
		Soybean by-products	2951.05	+0.09	+37.96	+5.04	−17.60
		Other soybean products	2327.69	+24.23	+77.72	−167.06	−22.28
Energy	Cocks	Corn	3321.82	+51.31	+ 39.42	−377.11	+113.92
		Corn by-products	4716.45	−227.63	+144.47	-	-
		Other Corn products	4133.39	−89.45	+100.32	−5.50	−96.37
Protein	Cocks	Soybean	4143.45	−3.18	−43.45	−213.55	+6.71
		Soybean by-products	518.54	+26.25	+47.10	+184.42	+69.0
		Other soybean products	6033.28	−15.02	−105.81	−556.50	+91.23

AMEn: nitrogen-corrected apparent metabolizable energy; CP: crude protein; EE: ether extract; CF: crude fiber.

The observed values (Alvarenga et al., 2011) and predicted (the result of the equations proposed by the BN) are plotted in the graph of Figure 4, and the statistics used in the assessment of the adjustment are shown in Table 3. The data used in this validation process coming from in vivo trials. Regarding the adjustments, the best evaluations of the statistics were MSE = 9051.84 for corn by-products, MAD = 81.66, MAPE = 2.16 and bias = −64.51 for other protein foods. The comparison between the predictions obtained in this research with the results of neural networks is shown in Table 4.

**Table 3.** Accuracy of prediction equations using data from in vivo trials (Alvarenga et al., 2011)

Equation	MSE	MAD	MAPE	bias
Corn	27314.73	143.28	3.98	−104.56
Soybean	71737.46	254.97	10.74	−254.97
Corn by-products	9051.84	82.85	3.44	81.26
Soybean by-products	131069.90	299.25	10.84	−227.12
Other energy food	64831.12	213.58	7.42	−184.02
Other protein food	16473.99	81.66	2.16	−64.51

MSE: mean squared error; MAD: mean absolute deviation; MAPE: mean absolute percentage error; PMSE: prediction mean squared error.

**Table 4.** Energy levels predicted from Bayesian networks (BN) and neural networks (NN, MARIANO et al., 2013) and bias found to result in vivo trials with chicks (Alvarenga et al., 2011)

		Prediction			
Food sample	Alvarenga et al. (2011)	BN	NN	BN^1^	NN^2^
Corn	3,747	3701.423	3682.410	**−45.577**	−64.590
	3,699	3718.852	3749.960	**19.852**	50.960
	3,813	3723.068	3691.330	**−89.931**	−121.670
	3,572	3783.902	3738.140	211.902	**166.140**
Soybean	2,373	2524.779	2532.720	**151.778**	159.720
	2,326	2703.916	2505.290	377.916	**179.290**
	2,355	2641.964	2500.180	286.963	**145.180**
	2,396	2677.614	2498.430	281.613	**102.430**
	2,478	2654.566	2513.110	176.566	**35.110**
Corn by-products	3,624	3628.778	3841.340	**4.777**	217.340
	3,676	3573.398	3803.920	**−102.601**	127.920
	2,184	2086.068	1931.880	**−97.432**	−251.620
Soybean by-products	3,159	3214.654	2527.200	**55.653**	−631.800
	3,779	3661.684	3580.770	**−117.315**	−198.230
	2,809	2992.756	2431.950	**183.756**	−377.050
	3,772	3938.579	3918.470	166.578	**146.470**
	2,387	2935.302	2342.300	548.302	**−44.700**
	3,971	3753.066	3519.050	**−217.934**	−451.950
	3,288	3617.693	3580.420	329.693	**292.420**
	2,314	2935.302	2351.800	621.302	**37.800**
	3,818	3753.066	3519.050	**−64.934**	−298.950
	3,173	3617.693	3580.420	444.693	**407.420**
	2,339	2935.302	2351.800	596.302	**12.800**
	3,793	3753.066	3519.050	**−39.934**	−273.950
	3,330	3617.693	3580.420	**287.693**	250.420
	2,309	2935.302	2351.800	626.302	**42.800**
	3,890	3753.066	3519.050	**−136.934**	−370.950
	3,267	3617.693	3580.420	350.693	**313.420**
Energy	3,598	3569.507	3498.87	**−28.492**	−99.130
	3,529	3515.214	3505.98	**−13.786**	−23.020
	3,862	3771.244	3537.47	**−90.756**	−324.530
	2,682	2839.403	2798.87	157.403	**116.870**
	1,941	2342.840	1939.12	401.840	**−1.880**
	3,362	3669.743	3512.24	307.492	**149.990**
Protein	3,934	3957.047	4049.53	**23.046**	115.53
	3,904	3979.133	4072.38	**74.880**	168.13

^1^BN: Prediction BN – Observed by Alvarenga et al. (2011); ^2^NN: Prediction NN – Observed by Alvarenga et al. (2011); Prediction NN: Obtained by Mariano et al. (2013).

## Discussion

This study aimed to propose and evaluate the use of BN and to find equations to the prediction of the AMEn values of energy and protein feedstuffs of vegetable origin used in the formulation of broiler rations. It is known, animal foods have quite different chemical compositions from vegetables, they have no fiber, soluble carbohydrates are extremely low, they have a high-fat content, and others. This variation in chemical and energy composition is even greater when it comes to animal by-products, due to the different processing methods and the lack of standardization of national products.

From this objective used machine learning algorithms to learn the graphic structure of the network as well as the probabilistic relationships between the variables, it was possible to prove the functionalities of this new promising methodology in broiler nutrition. The algorithm that showed the best performance was MMHC as the literature mentions in Felipe et al. (2015). It was observed that the equations differed in the values of the parameters due to countless DAG options (Koski and Noble, 2009). However, according to the lowest values of errors found in the validation using the test data (20%) in the Bayesian network model obtained the equations available in Table 3. In addition to the validation from the test data, the validation in the data of metabolic tests, only for chicks' equations, confirmed the efficiency of the obtained equations being indicated for the elaboration of balanced diets for broilers. The results continue to be proven through the predicted and realized values for AMEn, as shown below.

**Figure 3.**
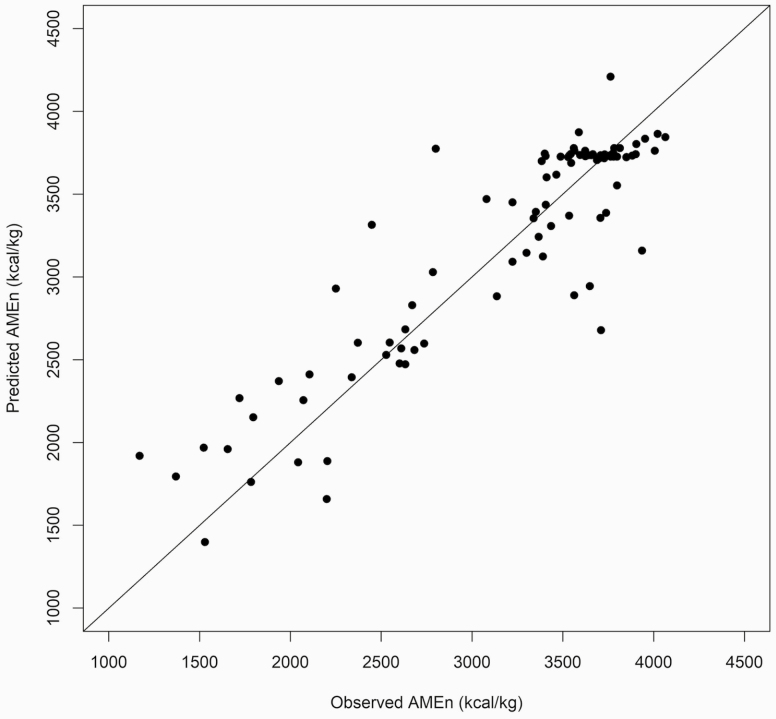
Relationship between the observed and predicted for nitrogen-corrected apparent metabolizable energy (AMEn) values of different feedstuffs using test data.

**Figure 4.**
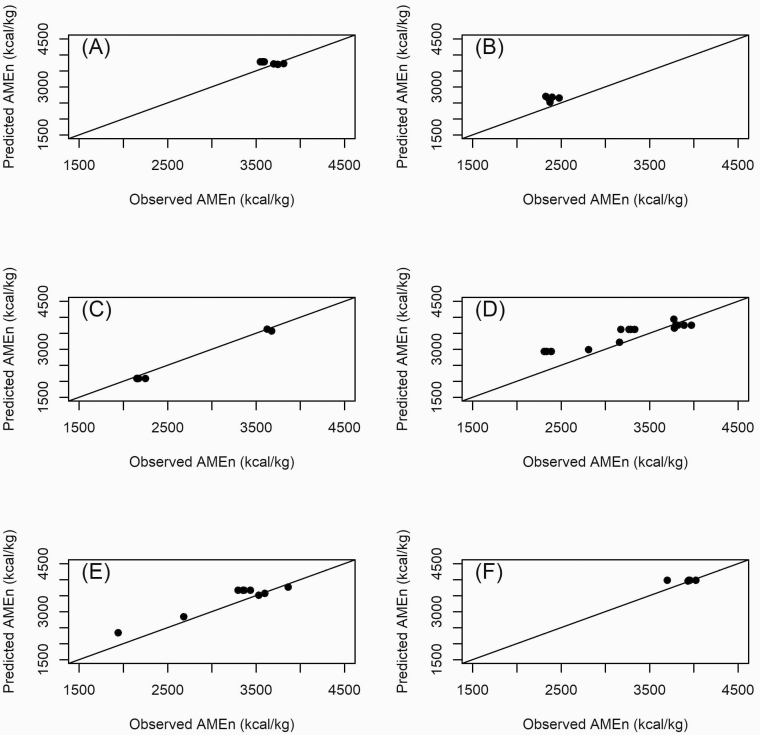
Predicted and realized values for nitrogen-corrected apparent metabolizable energy (AMEn) of different feedstuffs: corn (A), soybean (B), corn by-products (C), soybean by-products (D), other energy feeds (E), and other protein feeds (F) using data from chick in vivo trials (Alvarenga et al., 2011).

For comparison, in Nascimento et al. (2009), Mariano et al. (2012), and Mariano et al. (2013), the best architecture achieved *R*^2^ = 0.83, 0.74, and 0.86, respectively. In this research, the BN model managed to explain 87% of the AMEn variation. Predicted and realized values for AMEn are depicted in Figure 3. Errors in prediction, such as those we found, are attributed to the chemical composition of food in the ration considered (for some discussion on this, please refer to Moreira et al. (2002) and Brunelli et al. (2006). A metabolic trial was performed in chicks only. Thus, equations for cocks were not validated. Predictions and realizations based on data from (Alvarenga et al., 2011) are plotted in Figure 4. AMEn values are close to the identity line, indicating good accuracy of the proposed equations.

Equations proposed by the BN and those from the NN (Mariano et al., 2013) were validated with these in vivo trials with chicks (Alvarenga et al., 2011). The results can be found in Table 4. Predicted energy values that are closer to the realized description are described in boldface. From this table, we conclude that the BN predicted closer in 20 out of 36 cases and that the NN was closer in the other 16 cases. The equations for obtaining the energy values of corn, corn by-products, and other protein by means of BN had a better performance compared to the estimates obtained by NN (Mariano et al., 2013). These equations (Table 2) are for the corn (AMEn = 3658.16 − 2.41 CP − 11.25 EE + 83.41 ash + 16.76 CF), corn by-products (AMEn = 4209.57 − 34.56 CP + 32.84 EE − 25.15 ash − 142.57 CF) and others protein (AMEn = 2327.69 + 24.23 CP + 77.72 EE − 167.06 ash − 22.28 CF), the BN was remarkably better, but for soybeans, the opposite result was found.

It is known that the common statistical approach to obtain the AMEn values is that of ordinary least squares of multiple regression although there are few types of research of machine learning found for this purpose, these being NN. However, the authors advocate the use of computational methodologies, such as BN to predict AMEn and demonstrate that the use of BN for areas where machine learning and related methods are beginning to be employed; it has the benefits that traditional methods cannot achieve, especially the BN. BN capture conditional dependence between random variables in a broader sense and of relationships between discrete and continuous variables simultaneously in the model. Especially in the era of information, that computational methodologies have been experiencing have been more indicated by the listed properties. Emphasizes, to the AMEn values determined with chicks are found in Table 4, and that the values of AMEn for corn with BN (3,701.423 kcal/kg), NN (3,682.410 kcal/kg) and according to Rodrigues et al. (2001) using ordinary least squares of multiple regression, for the same feedstuff, the AMEn value was 3,699 kcal/kg, which declares the promising use of BN in bringing these values closer to the methods established by the literature.

According to the results found in this research, indicating good accuracy of the proposed equations via new machine learning methodology in poultry nutrition, authors in the literature show superiority in non-traditional models in the prediction of energy values. Ahmadi et al. (2007, 2008), Perai et al. (2010), and Mariano et al. (2013), demonstrated that the NN model outperformed the traditional models or accurately predicted performance based on dietary metabolizable energy.

The results demonstrated in Perai et al. (2010) that the NN model predicts the nitrogen-corrected true metabolizable energy (TMEn) values of meat and bone meat samples based on their chemical composition outperformed the traditional models. Accurately predicted metabolizable energy, methionine, and lysine using NN (Ahmadi et al., 2007) as well, predicted the TMEn values of feather and poultry offal meal based on their chemical composition (Ahmadi et al., 2008) are corroborant with the research and application of machine learning methods in poultry nutrition. In addition to Alvarenga et al. (2015) that reinforces innovations in estimation methods are necessary to obtain better estimates of the energy values of feed for broilers.

Felipe et al. (2015) compared different methodologies to predict total egg production in quails from different strains. The model with the combination of the BN and NN resulted in a better performance to predict total egg production. Töpner et al. (2017) used BN in a corn experiment to analyze the relationships between characteristics at genomic and residual levels. The BN obtained in this were classified in terms of adjustability and predictive ability through structural equations. They concluded that when illustrating the connections of characteristics concerning their genomic and residual nature, they become clearer, which makes it useful for predicting multiple traits and indirect selection. They confirm the potential of the BN in health sciences, economics, agriculture among others, that previously were unprecedented in the field of broiler nutrition.

In future studies, the dataset including other experimental studies will be updated. It will be to develop an innovative technological product based on the BN methodological proposal, with the objective of obtaining prediction equations to assist broiler nutritionists. Research the behavior of AMEn values in different probability distributions for the variables, to obtain prediction equations. Impute by BN the values of acid detergent fiber and neutral detergent fiber; missing variables or incomplete in the set data used and evaluate the effect of these values in the AMEn values. Increase the representativeness of the variables through the Bayesian Fuzzy Evolutionary Networks.

## CONCLUSIONS

After all, Alvarenga et al. (2015) have shown that these prediction equations are important for increasing the accuracy of diet formulation, allowing producers to correct energy values based on the variations in the chemical composition of feedstuffs. In conclusion, the MMHC algorithm and a partition with 80% of data to the training set seems to perform better in determining the DAG and respective BN. The BN was accurate and as good a method as the previous NN, depending on the food category. The predicting equations estimated from a BN can be used to calculate energy levels for broilers.
